# Crystal structure of a methanol solvate of a macrocycle bearing two flexible side-arms

**DOI:** 10.1107/S2056989021001067

**Published:** 2021-02-05

**Authors:** Felix Amrhein, Anke Schwarzer, Monika Mazik

**Affiliations:** a Technische Universität Bergakademie Freiberg, Leipziger Str. 29, D-09596 Freiberg/Sachsen, Germany

**Keywords:** crystal structure, macrocycle, hydrogen bonding and C—H⋯π inter­actions

## Abstract

Di-*tert*-butyl *N*,*N*′-{[13,15,28,30,31,33-hexa­ethyl-3,10,18,25,32,34-hexa­aza­penta­cyclo­[25.3.1.1^5,8^.1^12,16^.1^20,23^]tetra­triaconta-1(31),3,5,7,9,12(33),13,15,18,20,22,24,27,29-tetra­deca­ene-14,29-di­yl]bis­(methyl­ene)}dicarbamate methanol disolvate, C_52_H_72_N_8_O_4_·2CH_3_OH, was found to crystallize in the space group *P*2_1_/*c* with one half of the macrocycle (host) and one mol­ecule of solvent (guest) in the asymmetric unit of the cell, *i.e*. the host mol­ecule is located on a crystallographic symmetry center. Within the 1:2 host–guest complex, the solvent mol­ecules are accommodated in the host cavity and held in their positions by O—H⋯N and N—H⋯O bonds, thus forming ring synthons of graph set *R*_2_^2^(7). The connection of the 1:2 host-guest complexes is accomplished by C—H⋯O, C—H⋯N and C—H⋯π inter­actions, which create a three-dimensional supra­molecular network.

## Abstract 2

In the crystal structure of di-*tert*-butyl *N*,*N*′-{[13,15,28,30,31,33-hexa­ethyl-3,10,18,25,32,34-hexa­aza­penta­cyclo­[25.3.1.1^5,8^.1^12,16^.1^20,23^]tetra­triaconta-1(31),3,5,7,9,12 (33),13,15,18,20,22,24,27,29-tetra­deca­ene-14,29-di­yl]bis­(methyl­ene)}dicarbamate methanol disolvate, a pair of solvent mol­ecules is located in the cavity of the host mol­ecule.

## Chemical context   

Representatives of compounds consisting of a macrocyclic building block and two flexible side-arms have been shown to be able to act as powerful carbohydrate-binding agents (artificial carbohydrate receptors). Depending on the nature of their building blocks, various receptors with different binding properties could be developed (Lippe & Mazik, 2013 ▸, 2015 ▸; Amrhein *et al.*, 2016 ▸.). The design of such a receptor architecture was inspired by the results of our crystallographic studies, including the analyses of the binding motifs in complexes formed between acyclic receptors and monosaccharides, reported by us some time ago (Mazik *et al.*, 2005 ▸). At this point it should be noted that, in contrast to numerous known crystal structures of protein–carbohydrate complexes, there are only individual literature reports on the crystal structures of complexes formed between artificial receptors and sugars (for a recent report on such crystalline complexes, see Köhler *et al.*, 2020 ▸). The syntheses of the above-mentioned receptors, combining a macrocyclic building block and flexible side-arms, involve the preparation of macrocyclic precursors containing four imine functionalities. The crystal structure of one of such macrocyclic precursors is described in this work. This macrocycle bears two identical side-arms, containing the *tert*-butyl­oxycarbonyl group (BOC group), and is composed of two tri­ethyl­benzene units connected by two bridges, each bearing one pyrrole moiety and two imine functionalities.

## Structural commentary   

**Figure 1:**
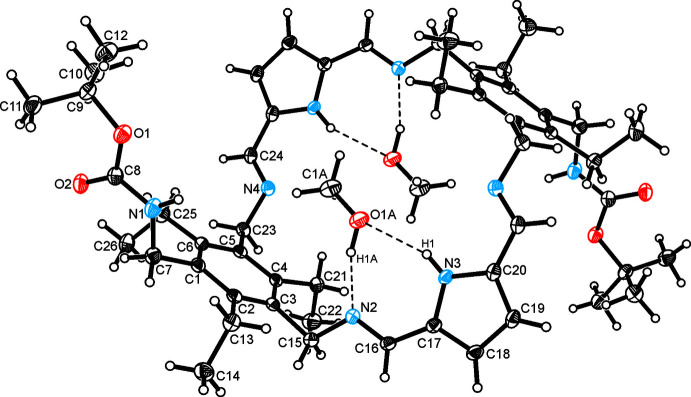
Perspective view of the 1:2 host–guest complex with methanol including the atom labeling. Anisotropic displacement ellipsoids are drawn at the 50% probability level.

The title compound was found to crystallize as a methanol solvate of the space group *P*2_1_/*c* with the asymmetric unit of the cell containing one half of the macrocycle and one solvent mol­ecule (the structure of the 1:2 host-guest complex is shown in Fig. 1 ▸), *i.e*. the host mol­ecule is located on a symmetry center. The bond lengths and angles confirm the expected structure and thus the presence of imino groups within the cyclic backbone [N2—C16 = 1.273 (2); N2—C15 = 1.478 (2); N4–C24 = 1.274 (2); N4—C23 = 1.463 (2) Å]. The substituents attached to the benzene ring adopt a fully alternating arrangement above and below the ring plane, *i.e*. the three ethyl groups all point in the opposite direction with regard to the pyrrole-based bridges connecting the two tri­ethyl­benzene units. The dihedral angle between the least-squares planes of the pyrrole and benzene rings is 76.0 (1)°, which corresponds with the torsion angles of 178.58 (12) and −131.22 (12)° for the atomic sequences C16—N2—C15—C3 and C24—N4—C23—C5, respectively. In the case of the side-arm bearing the BOC group the torsion angle along the atomic sequence C8—N1—C7—C1 amounts to 126.91 (14)°, whereas the torsion angles for the atom sequences C8—O1—C9—C10, C8—O1—C9—C11 and C8—O1—C9—C12 are −67.15 (15), 175.36 (12) and 57.39 (16)°.
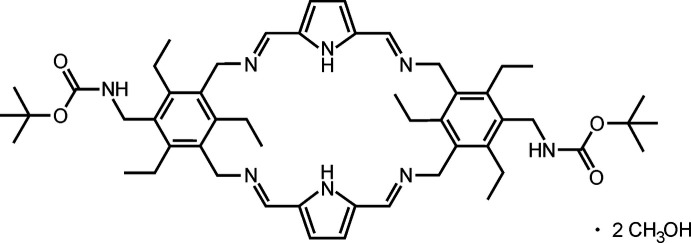


## Supra­molecular features   

**Figure 2:**
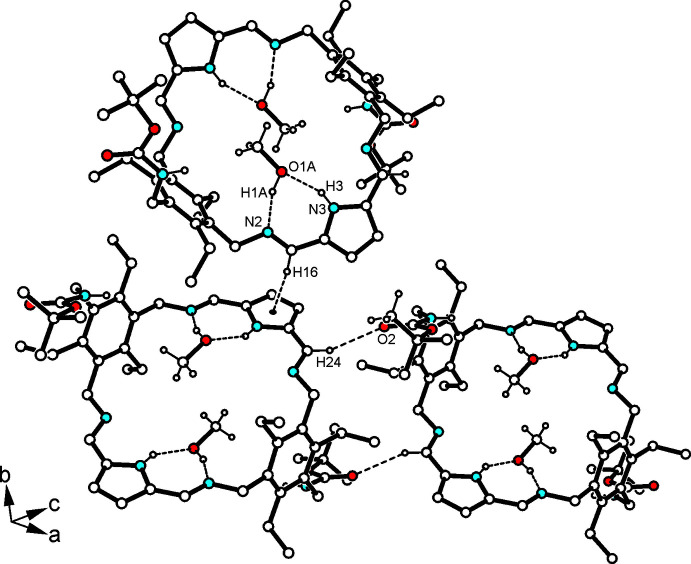
Part of the crystal structure of the 1:2 host–guest complex showing the mode of non-covalent inter­molecular bonding. For the sake of clarity, the H atoms of the host mol­ecule not involved in hydrogen-bonding inter­actions are omitted.

**Figure 3:**
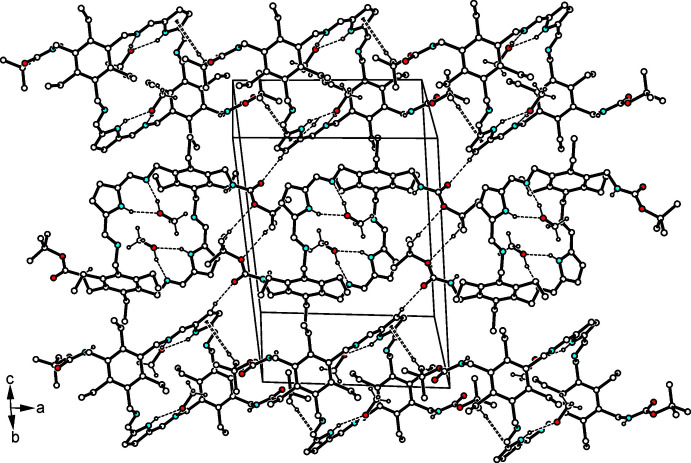
Packing diagram of the 1:2 host–guest complex. Hydrogen bonds and C—H⋯π inter­actions are represented by dashed lines and dashed double lines, respectively. For the sake of clarity, the H atoms of the host mol­ecule not involved in hydrogen-bonding inter­actions or C—H⋯π contacts are omitted.

Within the 1:2 host–guest complex, each of the methanol mol­ecules inter­acts with the host by a O—H⋯N_imine_ [*d*(H⋯N) = 1.82 (3) Å] and an N_pyrrole_-H⋯O hydrogen bond [*d*(H⋯O) = 2.10 (2) Å] that generate a cyclic synthon with a 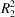
(7) motif according to Etter’s definition (Etter, 1990 ▸; Bernstein *et al.*, 1995 ▸). Thus, the hy­droxy group of each of the methanol mol­ecules participates in cooperative hydrogen bonds. The host–guest complexes are connected primarily by inter­actions involving the carbonyl oxygen atoms. Here, O2 acts as a bifurcated acceptor for the formation of C—H⋯O=C bonds [*d*(H⋯O) = 2.49, 2.52 Å], in which the imine atom H24 (see Figs. 2 ▸ and 3 ▸) and the pyrrole atom H18 of different mol­ecules are included. The second oxygen atom of the BOC group provides a weak C—H⋯O bond involving the *tert*-butyl group of the neighboring mol­ecule, which further participates in inter­molecular C—H⋯π inter­actions with the pyrrole unit of an adjacent host mol­ecule, as shown in Fig. 3 ▸ [*d*(H⋯*Cg*) = 3.00 Å]. In addition, the imine atom H16 contributes to formation of a C—H⋯π contact (see Fig. 2 ▸) with the pyrrole ring [*d*(H⋯*Cg*) = 2.88 Å]. The sum of these inter­actions creates a three-dimensional supra­molecular architecture. Numerical details are given in Table 1 ▸.

## Database survey   

A search of the Cambridge Structural Database (CSD, Version 5.41, update November 2019; Groom *et al.* 2016 ▸) for macrocyclic compounds containing two 2,4,6-tri­ethyl­benzene units and at least two pyrrole-based bridges connecting the two benzene rings gave four hits. They include multi-pyrrolic tripodal cages (ZOMPEZ; Wang *et al.*, 2019 ▸), a macrobicyclic cage (PEPGIB; Francesconi *et al.*, 2006 ▸), a hexa­mine macrobicycle with bound sulfate anion (ZOQCAL; Mateus *et al.*, 2015 ▸) as well as a macrobicycle with encapsulated phosphate ion (FOMBAN; Oh *et al.*, 2019 ▸). All four structures show an alternating orientation of the ring substituents.

## Synthesis and crystallization   

1-{[(1,1-Di­methyl­eth­oxy)carbon­yl]amino­meth­yl}-3,5-bis(amino­meth­yl)-2,4,6-tri­ethyl­benzene (Wiskur *et al.*, 2004 ▸) (172 mg, 0.50 mmol) was dissolved in dry ethanol (6 ml) and 1*H*-pyrrol-2,5-dicarboxaldehyde (61 mg, 0.50 mmol) was added. After the addition of a catalytic amount of acetic acid, the reaction mixture was stirred for 5 h at 318 K. The precipitated solid was filtered off, washed with small amount of dry ethanol and dried under vacuum. The product was obtained as a white solid (173 mg, 0.20 mmol, 81%). M.p. 533 K (decomp.); ^1^H NMR (500 MHz, CDCl_3_): *δ* = 1.17 (*t*, 6H, *J* = 7.5 Hz), 1.21 (*t*, 12H, *J* = 7.5 Hz), 1.38 (*s*, 18H), 2.57 (*q*, 8H, *J* = 7.5 Hz), 3.01–3.09 (*m*, 4H), 4.26 (*d*, 4H, *J* = 4.2 Hz), 4.36 (*s*, 2H), 4.72 (*br*, *s*, 8H), 6.51 (*s*, 4H), 8.22 (*s*, 4H), 9.54 (*s*, 2H) ppm. ^13^C NMR (125 MHz, CDCl_3_): *δ* = 15.01, 16.23, 22,43, 22.45, 28.39, 38.76, 57.97, 79.20, 114.11, 131.51, 132.85, 133.43, 142.74, 144.10, 151.10, 155.52 ppm; HRMS (ESI): C_52_H_72_N_8_O_4_ calculated for [*M* + H]^+^: 873.57493, found: 873.57663. Crystals suitable for single crystal X-ray diffraction were grown by slow evaporation of the solvent from the methanol solution of compound (I) at room temperature.

## Refinement   

Crystal data, data collection and structure refinement details are summarized in Table 2 ▸. The non-hydrogen atoms were refined anisotropically. The NH and OH hydrogens were located in a difference-Fourier map and refined freely. All other hydrogen atoms were positioned geometrically and allowed to ride on their parent atoms: C—H = 0.95 Å for imine and pyrrol H atoms, C—H = 0.99 Å for methyl­ene groups and C—H = 0.98 Å for methyl groups with *U*_iso_(H) = 1.5*U*_eq_(C) for methyl groups and *U*_iso_(H) = 1.2*U*_eq_(C) for other hydrogen atoms.

## Supplementary Material

Crystal structure: contains datablock(s) I, global. DOI: 10.1107/S2056989021001067/zq2258sup1.cif

Structure factors: contains datablock(s) I. DOI: 10.1107/S2056989021001067/zq2258Isup2.hkl

CCDC reference: 2059631

Additional supporting information:  crystallographic information; 3D view; checkCIF report

## Figures and Tables

**Table 1:** Hydrogen-bond geometry (Å, °) *Cg*2 represents the centroid of the C17–C20/N3 ring.

*D*—H⋯*A*	*D*—H	H⋯*A*	*D*⋯*A*	*D*—H⋯*A*
O1*A*—H1*A*⋯N2	0.96 (3)	1.82 (3)	2.7521 (16)	163 (2)
N3—H3⋯O1*A*	0.838 (18)	2.100 (18)	2.8757 (16)	153.6 (16)
C10—H10*C*⋯O1^i^	0.98	2.63	3.6094 (19)	173
C18—H18⋯O2^ii^	0.95	2.49	3.3345 (17)	148
C22—H22*A*⋯N4^iii^	0.98	2.73	3.6080 (18)	149
C24—H24⋯O2^iv^	0.95	2.52	3.4120 (17)	157
C25—H25*B*⋯O2	0.99	2.48	3.3988 (17)	154
C25—H25*B*⋯N1	0.99	2.58	3.3049 (19)	130
C12—H12*C*⋯*Cg*2^v^	0.98	3.00	3.7759 (18)	137
C16—H16⋯*Cg*2^vi^	0.95	2.88	3.7173 (15)	147

Symmetry codes: (i) -x, -y+1, -z+1; (ii) x+1, -y+{\script{1\over 2}}, z+{\script{1\over 2}}; (iii) -x+1, -y+1, -z; (iv) -x, -y+1, -z; (v) x-1, y, z; (vi) x, -y+{\script{1\over 2}}, z-{\script{1\over 2}}.

**Table 2:** Experimental details

Crystal data	
Chemical formula	C_52_H_72_N_8_O_4_·2CH_4_O
*M*_r_	937.26
Crystal system, space group	Monoclinic, *P*2_1_/*c*
Temperature (K)	100
*a*, *b*, *c* (Å)	13.8395 (9), 20.0443 (19), 9.6347 (9)
β (°)	102.800 (3)
*V* (Å^3^)	2606.3 (4)
*Z*	2
Radiation type	Mo *K*α
μ (mm^−1^)	0.08
Crystal size (mm)	0.35 × 0.31 × 0.21

Data collection	
Diffractometer	Bruker APEXII CCD
No. of measured, independent and observed [*I* > 2σ(*I*)] reflections	35355, 5099, 4185
*R*_int_	0.036
(sin θ/λ)_max_ (Å^−1^)	0.617

Refinement	
*R*[*F* ^2^ > 2σ(*F* ^2^)], *wR*(*F* ^2^), *S*	0.038, 0.103, 1.02
No. of reflections	5099
No. of parameters	326
H-atom treatment	H atoms treated by a mixture of independent and constrained refinement
Δρ_max_, Δρ_min_ (e Å^−3^)	0.32, −0.24

Computer programs: *APEX2* and *SAINT* (Bruker, 2007 ▸), *SIR2014* (Burla *et al.*, 2015 ▸), *SHELXL2018/3* (Sheldrick, 2015 ▸), *XP* (Sheldrick, 2008 ▸), *WinGX* (Farrugia, 2012 ▸), *publCIF* (Westrip, 2010 ▸) and *shelXle* (Hübschle *et al.*, 2011 ▸).

## supplementary crystallographic information

### Crystal data

**Table:**

C_52_H_72_N_8_O_4_·2CH_4_O	*F*(000) = 1016
*M**_r_* = 937.26	*D*_x_ = 1.194 Mg m^−^^3^
Monoclinic, *P*2_1_/*c*	Mo *K*α radiation, λ = 0.71073 Å
*a* = 13.8395 (9) Å	Cell parameters from 4018 reflections
*b* = 20.0443 (19) Å	θ = 2.6–30.1°
*c* = 9.6347 (9) Å	µ = 0.08 mm^−^^1^
β = 102.800 (3)°	*T* = 100 K
*V* = 2606.3 (4) Å^3^	Piece, colorless
*Z* = 2	0.35 × 0.31 × 0.21 mm

### Data collection

**Table:**

Bruker APEXII CCD diffractometer	*R*_int_ = 0.036
φ and ω scans	θ_max_ = 26.0°, θ_min_ = 3.1°
35355 measured reflections	*h* = −17→16
5099 independent reflections	*k* = −24→24
4185 reflections with *I* > 2σ(*I*)	*l* = −11→11

### Refinement

**Table:**

Refinement on *F*^2^	0 restraints
Least-squares matrix: full	Hydrogen site location: mixed
*R*[*F*^2^ > 2σ(*F*^2^)] = 0.038	H atoms treated by a mixture of independent and constrained refinement
*wR*(*F*^2^) = 0.103	*w* = 1/[σ^2^(*F*_o_^2^) + (0.049*P*)^2^ + 1.1359*P*] where *P* = (*F*_o_^2^ + 2*F*_c_^2^)/3
*S* = 1.02	(Δ/σ)_max_ < 0.001
5099 reflections	Δρ_max_ = 0.32 e Å^−^^3^
326 parameters	Δρ_min_ = −0.24 e Å^−^^3^

### Special details

**Table:**

Geometry. All esds (except the esd in the dihedral angle between two l.s. planes) are estimated using the full covariance matrix. The cell esds are taken into account individually in the estimation of esds in distances, angles and torsion angles; correlations between esds in cell parameters are only used when they are defined by crystal symmetry. An approximate (isotropic) treatment of cell esds is used for estimating esds involving l.s. planes.

### Fractional atomic coordinates and isotropic or equivalent isotropic displacement parameters (Å^2^)

**Table:**

	*x*	*y*	*z*	*U*_iso_*/*U*_eq_	
O1	−0.05242 (7)	0.39730 (5)	0.44678 (10)	0.0227 (2)	
O2	−0.09351 (7)	0.36986 (5)	0.21095 (10)	0.0239 (2)	
N1	0.05622 (9)	0.34295 (6)	0.35075 (14)	0.0234 (3)	
H1	0.0950 (13)	0.3465 (9)	0.437 (2)	0.033 (5)*	
N2	0.52052 (8)	0.30374 (6)	0.43228 (12)	0.0185 (3)	
N3	0.65652 (8)	0.34838 (6)	0.69247 (12)	0.0161 (2)	
H3	0.6091 (13)	0.3752 (9)	0.6721 (18)	0.026 (4)*	
N4	0.30737 (8)	0.53752 (6)	0.13277 (12)	0.0176 (2)	
C1	0.19144 (10)	0.33253 (7)	0.21758 (14)	0.0171 (3)	
C2	0.27938 (10)	0.29697 (7)	0.27053 (14)	0.0167 (3)	
C3	0.36923 (10)	0.32070 (7)	0.24387 (14)	0.0163 (3)	
C4	0.37244 (10)	0.38170 (7)	0.17394 (14)	0.0163 (3)	
C5	0.28382 (10)	0.41619 (7)	0.11840 (14)	0.0163 (3)	
C6	0.19328 (10)	0.39170 (7)	0.13994 (14)	0.0164 (3)	
C7	0.09242 (10)	0.30690 (7)	0.24027 (15)	0.0210 (3)	
H7A	0.042489	0.310462	0.149465	0.025*	
H7B	0.099383	0.259096	0.266375	0.025*	
C8	−0.03567 (10)	0.37006 (7)	0.32635 (15)	0.0196 (3)	
C9	−0.14526 (10)	0.43525 (7)	0.44325 (15)	0.0210 (3)	
C10	−0.14381 (12)	0.49841 (8)	0.35766 (17)	0.0267 (3)	
H10A	−0.205587	0.523136	0.352617	0.040*	
H10B	−0.137439	0.486846	0.261267	0.040*	
H10C	−0.087481	0.526152	0.403679	0.040*	
C11	−0.23587 (11)	0.39246 (8)	0.38596 (18)	0.0286 (4)	
H11A	−0.293766	0.412140	0.413275	0.043*	
H11B	−0.224754	0.347377	0.425669	0.043*	
H11C	−0.247562	0.390231	0.281954	0.043*	
C12	−0.13747 (12)	0.45120 (8)	0.59929 (16)	0.0293 (4)	
H12A	−0.195568	0.477016	0.609754	0.044*	
H12B	−0.077292	0.477311	0.635484	0.044*	
H12C	−0.134509	0.409572	0.653418	0.044*	
C13	0.27834 (11)	0.23254 (7)	0.35348 (15)	0.0214 (3)	
H13A	0.226010	0.235333	0.408604	0.026*	
H13B	0.342760	0.227356	0.421925	0.026*	
C14	0.25964 (12)	0.17120 (7)	0.25671 (17)	0.0271 (3)	
H14A	0.261688	0.130930	0.314968	0.041*	
H14B	0.310878	0.168419	0.201165	0.041*	
H14C	0.194388	0.174963	0.192008	0.041*	
C15	0.46409 (10)	0.28110 (7)	0.29158 (15)	0.0195 (3)	
H15A	0.447814	0.233201	0.296730	0.023*	
H15B	0.505554	0.286256	0.220681	0.023*	
C16	0.60090 (9)	0.27313 (7)	0.48501 (15)	0.0167 (3)	
H16	0.618598	0.236102	0.434316	0.020*	
C17	0.66663 (9)	0.29172 (6)	0.61784 (14)	0.0166 (3)	
C18	0.75267 (10)	0.25976 (7)	0.68732 (15)	0.0199 (3)	
H18	0.778419	0.219307	0.658912	0.024*	
C19	0.79463 (10)	0.29810 (7)	0.80710 (15)	0.0199 (3)	
H19	0.853574	0.288089	0.875574	0.024*	
C20	0.73443 (9)	0.35328 (7)	0.80740 (14)	0.0170 (3)	
C21	0.47064 (10)	0.41122 (7)	0.15895 (16)	0.0216 (3)	
H21A	0.523104	0.396530	0.240640	0.026*	
H21B	0.466421	0.460458	0.163095	0.026*	
C22	0.50054 (12)	0.39142 (8)	0.02052 (18)	0.0296 (4)	
H22A	0.565586	0.410479	0.019496	0.044*	
H22B	0.451306	0.408436	−0.061002	0.044*	
H22C	0.503828	0.342693	0.014619	0.044*	
C23	0.28733 (10)	0.48040 (7)	0.03607 (14)	0.0188 (3)	
H23A	0.339825	0.477093	−0.018671	0.023*	
H23B	0.223203	0.487046	−0.032276	0.023*	
C24	0.25124 (10)	0.58827 (7)	0.10195 (14)	0.0171 (3)	
H24	0.199470	0.587758	0.018667	0.021*	
C25	0.09723 (10)	0.42735 (7)	0.07478 (15)	0.0199 (3)	
H25A	0.110589	0.475578	0.067138	0.024*	
H25B	0.050286	0.422131	0.138050	0.024*	
C26	0.04981 (11)	0.39996 (8)	−0.07290 (16)	0.0262 (3)	
H26A	−0.013369	0.422704	−0.109625	0.039*	
H26B	0.038118	0.351973	−0.066133	0.039*	
H26C	0.094315	0.407676	−0.137487	0.039*	
O1A	0.45908 (8)	0.40005 (6)	0.59724 (13)	0.0377 (3)	
H1A	0.4690 (17)	0.3687 (12)	0.527 (3)	0.067 (7)*	
C1A	0.39245 (13)	0.44958 (9)	0.5307 (2)	0.0403 (4)	
H1AA	0.384837	0.483417	0.600927	0.060*	
H1AB	0.327948	0.429211	0.490507	0.060*	
H1AC	0.418223	0.470560	0.454430	0.060*	

### Atomic displacement parameters (Å^2^)

**Table:**

	*U*^11^	*U*^22^	*U*^33^	*U*^12^	*U*^13^	*U*^23^
O1	0.0201 (5)	0.0302 (6)	0.0185 (5)	0.0035 (4)	0.0054 (4)	0.0017 (4)
O2	0.0177 (5)	0.0318 (6)	0.0213 (5)	−0.0018 (4)	0.0021 (4)	−0.0011 (4)
N1	0.0182 (6)	0.0332 (7)	0.0186 (6)	0.0026 (5)	0.0037 (5)	0.0022 (5)
N2	0.0162 (6)	0.0177 (6)	0.0209 (6)	0.0009 (5)	0.0023 (5)	−0.0014 (5)
N3	0.0137 (5)	0.0144 (6)	0.0198 (6)	0.0022 (5)	0.0025 (5)	0.0023 (5)
N4	0.0176 (6)	0.0165 (6)	0.0187 (6)	−0.0022 (5)	0.0041 (5)	0.0010 (5)
C1	0.0167 (6)	0.0190 (7)	0.0159 (6)	−0.0021 (5)	0.0044 (5)	−0.0035 (5)
C2	0.0204 (7)	0.0160 (7)	0.0134 (6)	−0.0005 (5)	0.0031 (5)	−0.0022 (5)
C3	0.0171 (6)	0.0168 (7)	0.0141 (6)	0.0003 (5)	0.0017 (5)	−0.0042 (5)
C4	0.0153 (6)	0.0177 (7)	0.0160 (6)	−0.0019 (5)	0.0035 (5)	−0.0051 (5)
C5	0.0191 (7)	0.0157 (7)	0.0137 (6)	−0.0007 (5)	0.0029 (5)	−0.0031 (5)
C6	0.0160 (6)	0.0180 (7)	0.0147 (6)	0.0006 (5)	0.0022 (5)	−0.0031 (5)
C7	0.0194 (7)	0.0211 (7)	0.0234 (7)	−0.0015 (6)	0.0067 (6)	0.0011 (6)
C8	0.0181 (7)	0.0213 (7)	0.0205 (7)	−0.0035 (5)	0.0067 (6)	0.0028 (6)
C9	0.0172 (7)	0.0228 (7)	0.0240 (7)	0.0024 (6)	0.0069 (6)	0.0028 (6)
C10	0.0291 (8)	0.0247 (8)	0.0264 (8)	−0.0013 (6)	0.0065 (6)	0.0024 (6)
C11	0.0207 (7)	0.0263 (8)	0.0413 (9)	−0.0016 (6)	0.0124 (7)	−0.0009 (7)
C12	0.0296 (8)	0.0366 (9)	0.0241 (8)	0.0057 (7)	0.0107 (6)	0.0029 (7)
C13	0.0223 (7)	0.0211 (7)	0.0210 (7)	0.0006 (6)	0.0048 (6)	0.0041 (6)
C14	0.0307 (8)	0.0193 (7)	0.0310 (8)	−0.0022 (6)	0.0059 (7)	0.0015 (6)
C15	0.0191 (7)	0.0186 (7)	0.0200 (7)	0.0018 (5)	0.0028 (6)	−0.0026 (6)
C16	0.0160 (7)	0.0130 (6)	0.0226 (7)	−0.0007 (5)	0.0075 (5)	−0.0001 (5)
C17	0.0158 (6)	0.0133 (6)	0.0214 (7)	0.0003 (5)	0.0055 (5)	0.0024 (5)
C18	0.0185 (7)	0.0148 (7)	0.0268 (8)	0.0029 (5)	0.0055 (6)	0.0024 (6)
C19	0.0152 (6)	0.0193 (7)	0.0237 (7)	0.0011 (5)	0.0007 (5)	0.0056 (6)
C20	0.0144 (6)	0.0175 (7)	0.0188 (7)	−0.0021 (5)	0.0033 (5)	0.0047 (5)
C21	0.0167 (7)	0.0200 (7)	0.0285 (8)	−0.0033 (5)	0.0055 (6)	−0.0026 (6)
C22	0.0273 (8)	0.0273 (8)	0.0401 (9)	−0.0041 (6)	0.0198 (7)	−0.0029 (7)
C23	0.0196 (7)	0.0191 (7)	0.0172 (7)	−0.0012 (5)	0.0031 (5)	−0.0004 (5)
C24	0.0150 (6)	0.0190 (7)	0.0171 (7)	−0.0028 (5)	0.0032 (5)	0.0043 (5)
C25	0.0173 (7)	0.0198 (7)	0.0224 (7)	0.0019 (6)	0.0038 (5)	0.0030 (6)
C26	0.0203 (7)	0.0320 (9)	0.0241 (8)	0.0010 (6)	0.0000 (6)	0.0044 (6)
O1A	0.0296 (6)	0.0436 (7)	0.0353 (7)	0.0168 (5)	−0.0027 (5)	−0.0147 (6)
C1A	0.0345 (9)	0.0347 (10)	0.0494 (11)	0.0118 (8)	0.0046 (8)	−0.0090 (8)

### Geometric parameters (Å, º)

**Table:**

O1—C8	1.3481 (17)	C12—H12C	0.9800
O1—C9	1.4871 (16)	C13—C14	1.530 (2)
O2—C8	1.2176 (17)	C13—H13A	0.9900
N1—C8	1.3548 (18)	C13—H13B	0.9900
N1—C7	1.4636 (19)	C14—H14A	0.9800
N1—H1	0.886 (19)	C14—H14B	0.9800
N2—C16	1.2733 (17)	C14—H14C	0.9800
N2—C15	1.4780 (17)	C15—H15A	0.9900
N3—C20	1.3671 (17)	C15—H15B	0.9900
N3—C17	1.3679 (18)	C16—C17	1.4451 (19)
N3—H3	0.838 (18)	C16—H16	0.9500
N4—C24	1.2744 (17)	C17—C18	1.3868 (19)
N4—C23	1.4632 (17)	C18—C19	1.401 (2)
C1—C2	1.4052 (19)	C18—H18	0.9500
C1—C6	1.4054 (19)	C19—C20	1.3851 (19)
C1—C7	1.5239 (18)	C19—H19	0.9500
C2—C3	1.4071 (19)	C20—C24^i^	1.4486 (19)
C2—C13	1.5204 (19)	C21—C22	1.534 (2)
C3—C4	1.4014 (19)	C21—H21A	0.9900
C3—C15	1.5158 (18)	C21—H21B	0.9900
C4—C5	1.4065 (19)	C22—H22A	0.9800
C4—C21	1.5182 (18)	C22—H22B	0.9800
C5—C6	1.4033 (19)	C22—H22C	0.9800
C5—C23	1.5183 (19)	C23—H23A	0.9900
C6—C25	1.5173 (18)	C23—H23B	0.9900
C7—H7A	0.9900	C24—H24	0.9500
C7—H7B	0.9900	C25—C26	1.531 (2)
C9—C10	1.513 (2)	C25—H25A	0.9900
C9—C12	1.517 (2)	C25—H25B	0.9900
C9—C11	1.519 (2)	C26—H26A	0.9800
C10—H10A	0.9800	C26—H26B	0.9800
C10—H10B	0.9800	C26—H26C	0.9800
C10—H10C	0.9800	O1A—C1A	1.409 (2)
C11—H11A	0.9800	O1A—H1A	0.96 (3)
C11—H11B	0.9800	C1A—H1AA	0.9800
C11—H11C	0.9800	C1A—H1AB	0.9800
C12—H12A	0.9800	C1A—H1AC	0.9800
C12—H12B	0.9800		
			
C8—O1—C9	120.00 (11)	C13—C14—H14B	109.5
C8—N1—C7	122.09 (12)	H14A—C14—H14B	109.5
C8—N1—H1	118.8 (11)	C13—C14—H14C	109.5
C7—N1—H1	119.1 (12)	H14A—C14—H14C	109.5
C16—N2—C15	117.09 (11)	H14B—C14—H14C	109.5
C20—N3—C17	109.34 (11)	N2—C15—C3	111.32 (11)
C20—N3—H3	125.5 (12)	N2—C15—H15A	109.4
C17—N3—H3	125.2 (12)	C3—C15—H15A	109.4
C24—N4—C23	117.16 (11)	N2—C15—H15B	109.4
C2—C1—C6	120.32 (12)	C3—C15—H15B	109.4
C2—C1—C7	120.73 (12)	H15A—C15—H15B	108.0
C6—C1—C7	118.93 (12)	N2—C16—C17	123.50 (12)
C1—C2—C3	119.44 (12)	N2—C16—H16	118.2
C1—C2—C13	120.89 (12)	C17—C16—H16	118.2
C3—C2—C13	119.66 (12)	N3—C17—C18	107.86 (12)
C4—C3—C2	120.41 (12)	N3—C17—C16	124.20 (12)
C4—C3—C15	119.01 (12)	C18—C17—C16	127.81 (13)
C2—C3—C15	120.58 (12)	C17—C18—C19	107.40 (12)
C3—C4—C5	119.60 (12)	C17—C18—H18	126.3
C3—C4—C21	120.68 (12)	C19—C18—H18	126.3
C5—C4—C21	119.72 (12)	C20—C19—C18	107.46 (12)
C6—C5—C4	120.24 (12)	C20—C19—H19	126.3
C6—C5—C23	120.46 (12)	C18—C19—H19	126.3
C4—C5—C23	119.30 (12)	N3—C20—C19	107.93 (12)
C5—C6—C1	119.74 (12)	N3—C20—C24^i^	121.63 (12)
C5—C6—C25	120.21 (12)	C19—C20—C24^i^	130.17 (12)
C1—C6—C25	120.01 (12)	C4—C21—C22	113.67 (12)
N1—C7—C1	113.67 (12)	C4—C21—H21A	108.8
N1—C7—H7A	108.8	C22—C21—H21A	108.8
C1—C7—H7A	108.8	C4—C21—H21B	108.8
N1—C7—H7B	108.8	C22—C21—H21B	108.8
C1—C7—H7B	108.8	H21A—C21—H21B	107.7
H7A—C7—H7B	107.7	C21—C22—H22A	109.5
O2—C8—O1	125.71 (13)	C21—C22—H22B	109.5
O2—C8—N1	123.99 (13)	H22A—C22—H22B	109.5
O1—C8—N1	110.30 (12)	C21—C22—H22C	109.5
O1—C9—C10	108.95 (11)	H22A—C22—H22C	109.5
O1—C9—C12	102.34 (11)	H22B—C22—H22C	109.5
C10—C9—C12	110.92 (13)	N4—C23—C5	110.74 (11)
O1—C9—C11	111.04 (11)	N4—C23—H23A	109.5
C10—C9—C11	112.58 (12)	C5—C23—H23A	109.5
C12—C9—C11	110.54 (12)	N4—C23—H23B	109.5
C9—C10—H10A	109.5	C5—C23—H23B	109.5
C9—C10—H10B	109.5	H23A—C23—H23B	108.1
H10A—C10—H10B	109.5	N4—C24—C20^i^	120.67 (12)
C9—C10—H10C	109.5	N4—C24—H24	119.7
H10A—C10—H10C	109.5	C20^i^—C24—H24	119.7
H10B—C10—H10C	109.5	C6—C25—C26	111.82 (11)
C9—C11—H11A	109.5	C6—C25—H25A	109.3
C9—C11—H11B	109.5	C26—C25—H25A	109.3
H11A—C11—H11B	109.5	C6—C25—H25B	109.3
C9—C11—H11C	109.5	C26—C25—H25B	109.3
H11A—C11—H11C	109.5	H25A—C25—H25B	107.9
H11B—C11—H11C	109.5	C25—C26—H26A	109.5
C9—C12—H12A	109.5	C25—C26—H26B	109.5
C9—C12—H12B	109.5	H26A—C26—H26B	109.5
H12A—C12—H12B	109.5	C25—C26—H26C	109.5
C9—C12—H12C	109.5	H26A—C26—H26C	109.5
H12A—C12—H12C	109.5	H26B—C26—H26C	109.5
H12B—C12—H12C	109.5	C1A—O1A—H1A	108.8 (14)
C2—C13—C14	112.48 (12)	O1A—C1A—H1AA	109.5
C2—C13—H13A	109.1	O1A—C1A—H1AB	109.5
C14—C13—H13A	109.1	H1AA—C1A—H1AB	109.5
C2—C13—H13B	109.1	O1A—C1A—H1AC	109.5
C14—C13—H13B	109.1	H1AA—C1A—H1AC	109.5
H13A—C13—H13B	107.8	H1AB—C1A—H1AC	109.5
C13—C14—H14A	109.5		
			
C6—C1—C2—C3	−0.68 (19)	C7—N1—C8—O1	176.84 (12)
C7—C1—C2—C3	177.90 (12)	C8—O1—C9—C10	−67.15 (15)
C6—C1—C2—C13	−179.41 (12)	C8—O1—C9—C12	175.36 (12)
C7—C1—C2—C13	−0.82 (19)	C8—O1—C9—C11	57.39 (16)
C1—C2—C3—C4	4.51 (19)	C1—C2—C13—C14	88.14 (16)
C13—C2—C3—C4	−176.74 (12)	C3—C2—C13—C14	−90.59 (15)
C1—C2—C3—C15	−176.11 (12)	C16—N2—C15—C3	178.58 (12)
C13—C2—C3—C15	2.64 (18)	C4—C3—C15—N2	85.09 (15)
C2—C3—C4—C5	−6.00 (19)	C2—C3—C15—N2	−94.30 (14)
C15—C3—C4—C5	174.61 (12)	C15—N2—C16—C17	176.64 (12)
C2—C3—C4—C21	173.35 (12)	C20—N3—C17—C18	0.28 (15)
C15—C3—C4—C21	−6.04 (18)	C20—N3—C17—C16	−175.81 (12)
C3—C4—C5—C6	3.67 (19)	N2—C16—C17—N3	−8.3 (2)
C21—C4—C5—C6	−175.69 (12)	N2—C16—C17—C18	176.45 (14)
C3—C4—C5—C23	−176.83 (11)	N3—C17—C18—C19	0.42 (15)
C21—C4—C5—C23	3.82 (18)	C16—C17—C18—C19	176.32 (13)
C4—C5—C6—C1	0.12 (19)	C17—C18—C19—C20	−0.95 (16)
C23—C5—C6—C1	−179.38 (12)	C17—N3—C20—C19	−0.87 (15)
C4—C5—C6—C25	−177.44 (12)	C17—N3—C20—C24^i^	173.72 (12)
C23—C5—C6—C25	3.06 (19)	C18—C19—C20—N3	1.12 (15)
C2—C1—C6—C5	−1.61 (19)	C18—C19—C20—C24^i^	−172.86 (13)
C7—C1—C6—C5	179.77 (12)	C3—C4—C21—C22	92.25 (16)
C2—C1—C6—C25	175.95 (12)	C5—C4—C21—C22	−88.41 (16)
C7—C1—C6—C25	−2.66 (19)	C24—N4—C23—C5	−131.22 (12)
C8—N1—C7—C1	126.91 (14)	C6—C5—C23—N4	93.52 (14)
C2—C1—C7—N1	103.94 (15)	C4—C5—C23—N4	−85.98 (15)
C6—C1—C7—N1	−77.45 (16)	C23—N4—C24—C20^i^	179.56 (11)
C9—O1—C8—O2	−4.7 (2)	C5—C6—C25—C26	91.00 (15)
C9—O1—C8—N1	174.71 (11)	C1—C6—C25—C26	−86.55 (15)
C7—N1—C8—O2	−3.8 (2)		

Symmetry code: (i) −*x*+1, −*y*+1, −*z*+1.

### Hydrogen-bond geometry (Å, º)

*Cg*2 represents the centroid of the C17–C20/N3 ring.

**Table:**

*D*—H···*A*	*D*—H	H···*A*	*D*···*A*	*D*—H···*A*
O1*A*—H1*A*···N2	0.96 (3)	1.82 (3)	2.7521 (16)	163 (2)
N3—H3···O1*A*	0.838 (18)	2.100 (18)	2.8757 (16)	153.6 (16)
C7—H7*A*···O2	0.99	2.41	2.8234 (17)	104
C10—H10*C*···O1^ii^	0.98	2.63	3.6094 (19)	173
C10—H10*B*···O2	0.98	2.50	3.0916 (19)	119
C11—H11*C*···O2	0.98	2.41	2.8986 (18)	110
C18—H18···O2^iii^	0.95	2.49	3.3345 (17)	148
C22—H22*A*···N4^iv^	0.98	2.73	3.6080 (18)	149
C24—H24···O2^v^	0.95	2.52	3.4120 (17)	157
C25—H25*B*···O2	0.99	2.48	3.3988 (17)	154
C25—H25*B*···N1	0.99	2.58	3.3049 (19)	130
C12—H12*C*···*Cg*2^vi^	0.98	3.00	3.7759 (18)	137
C16—H16···*Cg*2^vii^	0.95	2.88	3.7173 (15)	147

Symmetry codes: (ii) −*x*, −*y*+1, −*z*+1; (iii) *x*+1, −*y*+1/2, *z*+1/2; (iv) −*x*+1, −*y*+1, −*z*; (v) −*x*, −*y*+1, −*z*; (vi) *x*−1, *y*, *z*; (vii) *x*, −*y*+1/2, *z*−1/2.
